# PROTOCOL: Service learning for improving academic success in students in grade K to 12: a systematic review

**DOI:** 10.1002/cl2.1157

**Published:** 2021-04-05

**Authors:** Trine Filges, Jens Dietrichson, Bjørn C. A. Viinholt, Nina T. Dalgaard

**Affiliations:** ^1^ VIVE‐The Danish Center for Social Science Research Copenhagen Denmark

## 1 BACKGROUND

### 1.1 Description of the condition

Completion of upper secondary education marks the minimum threshold for successful labour market entry and continued employability as suggested by the Organisation for Economic Co‐operation and Developments (OECD's) annual indicators on education and associated labour market outcomes (OECD, 2015). On average across OECD countries, unemployment risk of younger adults (25–34 year‐olds) who have not completed upper secondary education is almost double the risk of those with higher educational qualifications (upper secondary and postsecondary nontertiary education). A maintained focus on completion rates are necessary. Even though enrolment rates among 15–16 year olds (i.e., those typically in upper secondary programmes) are high; at least 95% on average across OECD countries in 2015 (OECD, 2018); far from all students graduate. According to OECD, only approximately 75% of students who had enroled had graduated after two years. Further, of the students who had not graduated, 80% were no longer enroled in education.

Many countries set specific targets for the completion rates of upper secondary education. For example, the countries in the European Union (EU) agreed on a 10‐year strategy proposed by the European Commission on March 3, 2010, for advancement of the economy of the EU (Europe, 2020). One of the main targets is to reduce the share of early school leavers to 10% from the (at that time) current 15% and increase the share of the population aged 30–34 having completed tertiary from 31% to at least 40% (European Commission, 2010). Some countries go even further as, for example, Denmark, setting as a specific target, that upper secondary completion rates should be 95% and tertiary enrolment and completion rates should be 60% by 2020 (OECD, 2013a).

Not only graduation rates are important, the quality of the education received also matters for the educational prospects of young people and successful entry into the labour market. The shares of neither employed nor in education or training (NEET) are negatively related to the skill levels among young people (OECD, 2017a). The OECD's Programme for International Student Assessment (PISA) tests students near the end of their compulsory education (usually around age 15) on their reading ability, their skills in math and level in sciences. In general, the higher the percentage of low‐performing 15‐year‐old students in PISA, the higher the percentage of NEETs among 15–19 year‐olds (OECD, 2017a).

Having acquired some of the knowledge and skills that are essential for full participation in modern societies, particularly in reading, mathematics and science may be more reliable predictors of economic and social well‐being than the number of years spent in school or in postformal education (OECD, 2016). Research based on the 2012 Survey of Adult Skills (PIAAC) finds that poor proficiency in numeracy and literacy limits access to rewarding and well‐paid jobs, and in addition is linked to poorer health and less social and political participation (OECD, 2013b).

There is, for these reasons, a significant interest in information about effective interventions to increase academic achievement and enhance educational prospects. The review we plan to conduct will focus on service learning in primary and secondary education. Service Learning is curriculum‐based community service that integrates classroom instruction with community service activities. The connection with specific courses and having clearly stated learning objectives is what distinguishes service learning from other forms of volunteer work. Service learning should “address real community needs in a sustained manner over a period of time; and assist students in drawing lessons from the service through regularly scheduled, organised reflection of critical analysis activities, such as classroom discussions, presentations, or directed writing” (Pritchard, 2002, p. 20). Well‐designed service‐learning activities can deepen learning and foster higher‐order thinking skills by providing students with opportunities to apply their learning to a challenging situation or problem in their community.

The development of service learning as a pedagogical method that integrates community service into the course curriculum began in the 1970s, primarily in the United States (Spring et al., 2008). In the nineties, service learning became institutionalised in public education in the United States (Peterson & Seligman, 2004). In 1990 in United States, the National and Community Service Act created Serve America (later named Learn and Serve America), which was a federal programme dedicated to providing grants and other supports for service learning activities in schools and community‐based organisations. Further, in 1994, service learning became a recognised method for meeting the aims of federal school funding (included in the Elementary and Secondary Education Act). In addition to these federal policies, several states and school districts mandated the incorporation of service learning into the course curriculum (Spring et al., 2008).

Service learning is not yet as widespread in the rest of the world. However, the OECD‐project “Innovative Learning Environments” mentions service learning as a pedagogical method to put learners at the centre (the first of the seven principles of learning needed to redesign the learning environments to meet the challenges of the 21^st^ century) (Dumont et al., 2010). According to Furco (2010) “service‐learning is one of the fastest growing educational initiatives in contemporary primary, secondary and post‐secondary education” (Furco, 2010, p. 228). Outside of the United States, service‐learning initiatives are part of the education systems of Argentina, Columbia and Singapore (Chua, 2010; Ierullo, 2016; Perold & Tapia, 2008). Argentina hosts the Latin American Center for Service‐Learning (CLAYSS) which was created in 2002 to support students, educators and community organisations in the development of service‐learning projects in Latin America. Service learning is not part of any educational policy in Europe although the EU recognises service learning as a way of achieving citizenship education (European Commission/EACEA/Eurydice, 2017). Service learning is however emerging in many European countries including Germany, Ireland, Italy, Spain and the United Kingdom (Furco, 2010) and currently CLAYSS is assisting in the creation of the Central and Eastern European Service‐Learning Network (Regina & Ferrara, 2017).

In several European nations there are organisations (nonprofit community‐based) with programmes dedicated to providing supports for service learning activities in schools (Luna, 2012): Lernen durch Engagement in Germany, Center for Frivilligt Socialt Arbejde in Denmark, Lernen durch Engagement in Switzerland, Noi‐orizonturi in Romania, MOVISIE in the Netherlands and Fundación Tomillo in Spain.

### 1.2 Description of the intervention

School‐based service‐learning is a teaching strategy that explicitly links community service to academic instruction (Billig, 2000). In the United States, “service‐learning” is an official term used by policy makers and educational leaders. Service‐learning is distinctive from traditional voluntarism or community service in that it intentionally connects service activities with curriculum concepts and includes structured time for reflection. Service‐learning is not an add‐on to an existing curriculum, a requirement of a minimum hours of service to graduate or service assigned as punishment. Rather, students are required to use academic knowledge and skills to address genuine community needs. A clarifying example is given by the National Youth Leadership Council (https://www.nylc.org/page/our-philosophy):

“Picking up trash on a river bank is service.

Studying water samples under a microscope is learning.

When science students collect and analyze water samples, document their results and present findings to a local pollution control agency—that is service‐learning”.

Service learning programmes can take many forms and are very diverse in content. However, a common set of elements are critical for a success full implementation of service learning. The National Youth Leadership Council and RMC Research Associates have developed a set of eight quality service‐learning standards (the K‐12 Service‐Learning Standards for Quality Practice) with input from youth, teachers, administrators, youth agencies, policymakers, community members and other stakeholders. The standards are:

- **Meaningful service:** Service‐learning actively engages participants in meaningful and personally relevant service activities.
- **Link to curriculum:** Service‐learning is intentionally used as an instructional strategy to meet learning goals and/or content standards.
- **Reflection:** Service‐learning incorporates multiple challenging reflection activities that are ongoing and that prompt deep thinking and analysis about oneself and one's relationship to society.
- **Diversity:** Service‐learning promotes understanding of diversity and mutual respect among all participants.
- **Youth voice:** Service‐learning provides youth with a strong voice in planning, implementing and evaluating service‐learning experiences with guidance from adults.
- **Partnerships:** Service‐learning partnerships are collaborative, mutually beneficial and address community needs.
- **Progress monitoring:** Service‐learning engages participants in an ongoing process to assess the quality of implementation and progress toward meeting specified goals and uses results for improvement and sustainability.
- **Duration and intensity:** Service‐learning has sufficient duration and intensity to address community needs and meet specified outcomes.

The complete document can accessed at https://www.nylc.org/page/standards.

### 1.3 How the intervention might work

Research shows that the students who participate in service learning may benefit both personally, socially and academically (e.g., Celio et al., 2011; RMC Research Corporation, 2002). Service learning, by connecting education to real world issues and allowing students to address problems they identify, may be particularly efficacious as it increases engagement and motivates students, in particular students who might not respond well to more traditional teaching methods (see, e.g., Bridgeland et al., 2008; Kraft & Wheeler, 2003; Scales & Roehlkepartain, 2005).

Motivation for learning and school engagement play a critical role in students' academic success (e.g., Fan & Wolters, 2014; Skaalvik & Valas, 1999). Motivated students tend to do better at school. According to OECD, students who are among the most motivated score the equivalent of more than one school year higher in PISA than the least‐motivated students and motivation is further positively related to life satisfaction (OECD, 2017b).

Theoretically, Kolb's (1984) model of experiential learning is often referred to as the foundation for understanding how service‐learning might work. Experiential learning theory defines learning as “The process whereby knowledge is created through the transformation of experience” and knowledge is defined as: “a transformation process being continuously created and recreated, not an independent entity to be acquired or transmitted” (Kolb, 1984, p. 38). Kolb further suggests that experiential approaches to learning such as service‐learning are better at accommodating learners with different learning styles than traditional didactic approaches such as classroom‐based teaching. Experiential learning is inspired by pragmatist philosopher John Dewey's six‐step process of experiential logical inquiry. According to Dewey the six steps are: (1) encountering a problem, (2) formulating a problem or question to be resolved, (3) gathering information which suggests solutions, (4) making hypotheses, (5) testing hypotheses and (6) making warranted assertions (Dewey, 1938; Giles & Eyler, 1994; Kolb,^1984^). Kolb's (1984) model comprises these steps into a four stage experiential learning cycle involving: *Concrete Experiences, Reflective Observation, Abstract Conceptualisation* and *Active Experimentation* (Cone & Harris, 1996; Kolb, 1984). Based on this conception, students participating in service‐learning are engaged in a cycle in which their work in the community promotes written and/or oral reflection. Under the guidance of teachers or instructors, reflective work may be used to form abstract concepts and generate hypotheses, which may then be cycled back into further concrete experiences. According to Kolb this way of learning allows a variety of students with different learning styles and abilities to develop and integrate their skills (Cone & Harris, 1996). Service‐learning provides an opportunity for students to move between perceiving new information through experiencing the concrete, tangible, felt qualities of the world within the community and taking hold of new information through abstract conceptualisation, thinking and analyzing. The pattern in which a learner moves between these levels of experience are thought to reflect an individual learning style, and service‐learning is thought to allow each student to move between the levels in a way consistent with their own learning style (Kolb et al., 2002).

Another strand of theory which offers a potential understanding of the theory of change behind service‐learning is *Situated Learning*. The term “situated learning” refers to learning that occurs within a particular and authentic context through the individual's social participation. Rather than focusing on learning as a primarily cognitive process involving a number of tasks, situated learning theorists study the process in which individuals become new members of a learning community. According to the theory newcomers within a learning community move from a state of legitimate peripheral participation to full participation through a process that involves continuous negotiation, collaboration and reflection (Wolfson & Willinsky, 1998).

In their often cited work: “Situated Learning: Legitimate Peripheral Participation”, Lave and Wenger (1991) focus on acquisition of skills and knowledge that takes place outside of traditional schooling within communities of practice. Based on an ethnographic investigation of traditional and nontraditional apprenticeships in Mexico, Liberia and the United States, Lave and Wenger propose that learning should not be viewed as the mere transmission of knowledge but as a distinctly embedded and active process. Learning is thus perceived as a contextualised process in which content is learned through doing activities. Furthermore, Lave and Wenger suggest that motivation too is “situated”, as learners are naturally motivated by their growing value of participation (Lave & Wenger, 1991). Based on this approach students participating in service‐learning inherently become motivated to learn as this enables them to move from being novices to becoming full participants within the learning community. Furthermore, students participating in service‐learning may become motivated as they experience how their own participation increases in value as they progress from being newcomers towards the centre of the community of practice.

In situated learning the construction of meaning is seen as being tied to specific contexts and purposes. For students participating in service‐learning this may be particularly important, as service learning may enable them to socially construct meaning which makes learning matter beyond school.

### 1.4 Why it is important to do this review

Two systematic reviews with meta‐analyses are found in Conway et al. (2009) and Celio et al. (2011), both performing searches up to spring 2008. The review by Conway et al. (2009) analysed four outcomes: academic, personal, social and citizenship outcomes. They included studies of community service or volunteerism as well as service learning without distinguishing between these very different types of interventions (except in a moderator analysis), participants were not limited to primary and secondary education (although all results were shown separately for grade kindergarten to 12 students but without distinguishing between community service or volunteerism and service learning) and many of the included studies did not have control groups.

The review by Celio et al. (2011) required included studies to analyse service learning using a control group but participants were not limited to primary and secondary education. Five outcome areas were analysed: attitudes toward self, attitudes toward school and learning, civic engagement, social skills and academic achievement. Separate results for primary and secondary education (grades kindergarten to 12) was only shown for the overall effect, that is, the mean of the five outcomes attitudes toward self, attitudes toward school and learning, civic engagement, social skills and academic achievement. The analysis of primary and secondary education outcomes did not take into consideration that more than one outcome per study was included in the meta‐analysis (i.e., they did not take into account the dependencies between the effect sizes).

Besides being up to date, the major differences between these two systematic reviews and the current proposal are that we will focus on service learning for primary and secondary education, only include studies with a control group, all relevant outcomes areas will be analysed separately and we will take into consideration the dependencies between effect sizes.

In addition, there are several literature reviews of studies conducted in the United States (Billig, 2000, 2002, 2003, 2004). None of them is a systematic review and no data synthesis is performed in any of them. The review we plan to do differs in substantial ways from these existing reviews. It is systematic and a meta‐analysis will be conducted.

## 2 OBJECTIVES

The main objective of this review is to answer the following research question: What are the effects of service learning on academic success, NEET status, personal and social skills and risk behaviour of students in primary and secondary education (grades kindergarten to 12)?

Further, we will investigate the following factors with the aim of explaining potential observed heterogeneity: study‐level summaries of participant characteristics (e.g., studies considering a specific gender, age or socioeconomic level or studies where separate effects for girls/boys, primary school/secondary school or low/high socioeconomic status are available) and quality of the service learning programme according to the standards as outlined in section *The intervention*. The moderator analysis will be performed as outlined in section *Moderator analysis and investigation of heterogeneity*.

## 3 METHODS

### 3.1 Criteria for considering studies for this review

#### 3.1.1 Types of studies

The proposed project will follow standard procedures for conducting systematic reviews using meta‐analysis techniques.

Randomised controlled trials will be included. In order to summarise what is known about the possible causal effects of service learning, we will include all study designs that use a control group, that is, a group of students not participating in service learning. The control group may be offered treatment as usual or an alternative treatment.

The study designs we will include in the review are:

- Randomised and quasi‐randomised controlled trials (allocated at either the individual level or cluster level, e.g., class/school/geographical area etc.).
- Nonrandomised studies (service learning has occurred in the course of usual decisions, the allocation to service learning and no service learning is not controlled by the researcher, and there is a comparison of two or more groups of participants, i.e., at least a treated group and a control group).

Studies using single group pre‐post comparisons will not be included. Nonrandomised studies using an instrumental variable approach will not be included—see the Appendix (*Justification of exclusion of studies using an instrumental variable (IV) approach*) for our rationale for excluding studies of these designs. A further requirement to all types of studies (randomised as well as nonrandomised) is that they are able to identify an intervention effect. Studies where, for example, the treatment is given to teachers in one school only and the comparison group is teachers at another school (or more schools for that matter) cannot separate the treatment effect from the school effect. Even within schools, organisation of teachers in teacher teams may mean that randomisation would have to be at the teacher team level to be able to avoid a situation of not being able to separate teacher‐level treatment effect from teacher‐team effect.

#### 3.1.2 Types of participants

The review will include children in primary and secondary education (grades kindergarten to 12) in general education.

The included grades correspond to primary and secondary school, defined as the first two steps in a three‐tier educational system consisting of primary education, secondary education and tertiary or higher education. The number of years a child attend primary schooling varies across the OECD countries, though most often primary schooling is K‐6 or K‐9 after which secondary education begins (e.g., in the form of high school). The former is the case for instance in France, Spain, Japan, UK and most parts of Australia, and the second is the case for school systems in countries such as Italy, Turkey, Sweden and Denmark. The age range included will differ between countries, and sometimes between states within countries. Typically, ages range from 5–7 to 11–13. In some countries, kindergarten can however refer to preschool programmes outside of primary school and include ages down to 2 years. Service learning targeting such populations will be excluded; that is, kindergarten must be considered a part of primary school for a study to be included.

Grades 7–12 corresponds roughly to secondary school, defined as the second step in a three‐tier educational system. The number of years a child attend secondary schooling varies across the OECD countries, though most often secondary schooling is grades 7–12 or 10–12. The former is the case for instance in France, Spain, Japan, UK and most parts of Australia, and the second is the case for school systems in countries such as Italy, Turkey, Sweden and Denmark. The age range included will differ between countries, and sometimes between states within countries. Typically, ages will range from 12–14 to 17–19.

Studies that meet inclusion criteria will be accepted from all countries. We will exclude children in home school and in preschool programmes.

#### 3.1.3 Types of interventions

Service Learning is a curriculum‐based community service that integrates classroom instruction (such as classroom discussions, presentations, or directed writing) with community service activities. Service learning may be mandatory or voluntary, and should have service activities that take place outside of the classroom. It should take place in the community including the school as part of the community. Service learning is organised in relation to an academic course or curriculum and has clearly stated learning objectives. Service learning should address real community needs and involve students in drawing lessons from the service through regularly scheduled, organised reflection or critical analysis. Community service or extracurricular activities that do not integrate classroom instruction will be excluded.

#### 3.1.4 Types of outcome measures

##### Primary outcomes

The primary focus is on measures of academic success and NEET status (neither employed nor in education or training post compulsory school). The primary outcomes are:

- Scores on students' achievement tests
- Attendance
- Drop‐out
- Eemployment, education, training (NEET status)

Concerning scores on students' achievement tests, only standardised measures will be included, that is, norm‐referenced tests (e.g., Gates‐MacGinitie Reading Tests and Star Math), state‐wide tests (e.g., Iowa Test of Basic Skills), national tests (e.g., National Assessment of Educational Progress) and measures of global academic performance (e.g., Woodcock‐Johnson III Tests of Achievement, Stanford Achievement Test (SAT), Grade Point Average).

Although we do not expect to find studies reporting follow up outcomes in the long run (post compulsory school), NEET status is included as a primary outcome.

##### Secondary outcomes

A secondary focus is on measures of personal and social skills (including self‐perception/self‐confidence and attitudes towards helping others) and risk behaviour (such as drug and alcohol use, violent behaviour, sexual risk taking; measured by self‐reports or reports by authorities, administrative files, registers).

Concerning personal and social skills, only valid and reliable outcomes that have been standardised on a different population (and is “objective,” i.e., not “experimenter‐designed”) will be included. Examples of valid outcomes are measures from the Social Skills Rating System (SSRS; Gresham & Elliott, 1990) or the revision of the SSRS, called the Social Skills Improvement System‐Rating Scales (SSIS‐RS; Gresham & Elliott, 2008) and the Academic Competence Evaluation Scales (ACES) (DiPerna & Elliott, 1999).

Studies will only be included if they consider at least one of the primary or secondary outcomes. If it is not clear from the description of outcome measures in the studies whether they are standardised, we will use electronic sources to determine whether a measure is standardised or not. We will not consider measures where researchers have picked a subset of questions from a standardised measure.

It will be reported if any potential adverse effects have been evaluated in any included studies.

**Duration of follow‐up**

Time points for measures considered will be:

- 0–1 year follow up
- 1–2 year follow up
- More than 2 year follow up

**Types of settings**

The location of the intervention is classes, primary and secondary education (grades kindergarten to 12) in regular private, public or boarding schools. Home‐schools will be excluded.

### 3.2 Search methods for identification of studies

Relevant studies will be identified through searches in electronic databases, grey literature repositories and resources, hand search in specific targeted journals, citation tracking, contact to international experts and internet search engines.

#### 3.2.1 Electronic searches

Following bibliographic databases will be searched:

- SocINDEX
- PsycINFO
- EconLit
- ERIC
- Academic Search
- Science Citation Index
- Social Science Citation Index
- Sociological Abstracts
- CINAHL
- Teacher Reference Center
- Cochrane Library

An example of the search strategy used for the databases on the EBSCO‐host platform is listed below:

**Table:** 

Terms	Search
S9	S7 AND S8
S8	S4 OR S5 OR S6
S7	S1 OR S2 OR S3
S6	(AB school* OR AB communit*) AND (AB student* OR AB pupil* OR AB adolescen*)
S5	AB student* OR AB pupil* OR AB adolescen*
S4	TI student* OR TI pupil* OR TI school* OR TI adolescen*
S3	AB "service learning"
S2	TI "service learning"
S1	DE "Service Learning"

The search string will be modified to match the subject terms and search interface of the different databases.

#### 3.2.2 Searching other resources

**Grey literature resources**

Following grey literature resources will be searched:

- ProQuest Dissertations & Theses Global
- EBSCO Open Dissertations
- Danish National Research Database—http://www.forskningsdatabasen.dk/en
- SSRN Working Papers—http://www.ssrn.com
- Open Grey—http://opengrey.eu/
- Google Scholar—https://scholar.google.com
- Google searches—https://google.com/
- Education Commission of the States: https://www.ecs.org/
- National Youth Leadership Council: https://www.nylc.org/
- Search Institute: https://www.search-institute.org/
- Manpower Demonstration Research Corporation: https://www.mdrc.org/
- American Institutes for Research: https://www.air.org/
- RAND: https://www.rand.org/
- Mathematica: https://mathematica.org/
- CIRCLE (The Center for Information and Research on Civic Learning and Engagement): https://civicyouth.org/ResearchTopics/research-topics/service-learning/

Further sources of grey literature might be added throughout the search process.

**Hand search**

Seven specific journals will be hand‐searched:

- International Journal for Research on Service‐Learning and Teacher Education
- Journal of Experiential Education
- Journal of Adolescence
- Journal of Early Adolescence
- Journal of Prevention and Intervention in the Community
- Advances in Service‐Learning Research Series
- The International Journal of Research on Service‐Learning and Community Engagement

**Citation tracking**

In order to identify both published studies and grey literature we will utilise citation‐tracking/snowballing strategies. Our primary strategy will be to citation‐track related systematic‐reviews and meta‐analyses. The review team will also check reference lists of included primary studies for new leads.

**Contact with international experts**

We will contact international experts to identify unpublished and ongoing studies.

### 3.3 Data collection and analysis

#### 3.3.1 Description of methods used in primary research

Randomised controlled trials are eligible but we expect that a certain amount of studies will be conducted without randomisation of participants. Studies of the effect of service learning are required to have a control group for inclusion in the review. Participants may be allocated by, for example, time differences, location differences, decision makers, policy rules or participant preferences. They must all demonstrate pretreatment group equivalence via matching, statistical controls, or evidence of equivalence on key risk variables and participant characteristics. The methodological appropriateness will be assessed according to the risk of bias model outlined in section “Assessment of risk of bias in included studies”. The risk of bias assessment makes it possible to discriminate between studies with varying degrees of risk. Studies that have been coded with a Critical risk of bias will not be included in the data synthesis.

An example of a study that may be included is O'Donell et al. (1999), in which students at one school were randomly assigned by classroom to receive either a Reach for Health classroom curriculum or a Reach for Health service learning programme. Another study, Scales et al. (2000), randomly assigned students in three schools to teams, where after schools determined which of their teams would be service‐learning teams and which would be control teams. A series of analysis of covariances (ANCOVAs) were conducted to compare service‐learning students with control students with pre‐test scores on the dependent variables as the covariates. A third example is the study by Melchior (1998) which reports on the conduct and findings of a 3‐year evaluation of the 1995–1996 school year of the Learn and Serve America School and Community‐Based Programmes. Service learning participants were compared with students in similar types of classes in the same schools, matched as closely as possible with participants in terms of demographic characteristics (age, gender, race/ethnicity, etc.) and academic status. The author further applies two different statistical control methods (ANCOVA and difference‐in‐difference) in order to adjust for baseline differences.

#### 3.3.2 Selection of studies

Under the supervision of review authors, two review team assistants will first independently screen titles and abstracts to exclude studies that are clearly irrelevant. Studies considered eligible by at least one assistant or studies were there is insufficient information in the title and abstract to judge eligibility, will be retrieved in full text. The full texts will then be screened independently by two review team assistants under the supervision of the review authors. Any disagreement of eligibility will be resolved by the review authors. Exclusion reasons for studies that otherwise might be expected to be eligible will be documented and presented in an appendix.

The study inclusion criteria will be piloted by the review authors (see Appendix *First and second level screening*). The overall search and screening process will be illustrated in a flow diagram. None of the review authors will be blind to the authors, institutions, or the journals responsible for the publication of the articles.

#### 3.3.3 Data extraction and management

Two review authors will independently code and extract data from included studies. A coding sheet will be piloted on several studies and revised as necessary (see Appendix *Data extraction*). Disagreements will be resolved by consulting a third review author with extensive content and methods expertise. Disagreements resolved by a third reviewer will be reported. Data and information will be extracted on: available characteristics of participants, intervention characteristics and control conditions, research design, sample size, risk of bias and potential confounding factors, outcomes and results. Extracted data will be stored electronically. Analysis will be conducted using RevMan5 and Stata software.

#### 3.3.4 Assessment of risk of bias in included studies

We will assess the risk of bias in randomised studies using Cochranes revised risk of bias tool, ROB 2 (Higgins et al., 2019).

The tool is structured into five domains, each with a set of signalling questions to be answered for a specific outcome. The five domains cover all types of bias that can affect results of randomised trials.

The five domains for individually randomised trials are:

(1) Bias arising from the randomisation process;
(2) Bias due to deviations from intended interventions (separate signalling questions for effect of assignment and adhering to intervention);
(3) Bias due to missing outcome data;
(4) Bias in measurement of the outcome;
(5) Bias in selection of the reported result.

For cluster‐randomised trials, an additional domain is included ((1b) Bias arising from identification or recruitment of individual participants within clusters). We will use the latest template for completion (currently it is the version of March 15, 2019, for individually randomised parallel‐group trials and October 20, 2016, for cluster randomised parallel‐group trials). In the cluster randomised template, however, only the risk of bias due to deviation from the intended intervention (effect of assignment to intervention; intention to treat) is present and the signalling question concerning the appropriateness of the analysis used to estimate the effect is missing. Therefore, for cluster randomised trials we will only use the signalling questions concerning the bias arising from identification or recruitment of individual participants within clusters from the template for cluster randomised parallel‐group trials; otherwise we will use the template and signalling questions for individually randomised parallel‐group trials.

We will assess the risk of bias in nonrandomised studies, using the model ROBINS‐I, developed by members of the Cochrane Bias Methods Group and the Cochrane Non‐Randomised Studies Methods Group (Sterne, Higgins, et al., 2016). We will use the latest template for completion (currently it is the version of September 19, 2016).

The ROBINS‐I tool is based on the Cochrane RoB tool for randomised trials, which was launched in 2008 and modified in 2011 (Higgins et al., 2011).

The ROBINS‐I tool covers seven domains (each with a set of signalling questions to be answered for a specific outcome) through which bias might be introduced into nonrandomised studies:

(1) Bias due to confounding
(2) Bias in selection of participants
(3) Bias in classification of interventions
(4) Bias due to deviations from intended interventions;
(5) Bias due to missing outcome data;
(6) Bias in measurement of the outcome;
(7) Bias in selection of the reported result.

The first two domains address issues before the start of the interventions and the third domain addresses classification of the interventions themselves. The last four domains address issues after the start of interventions and there is substantial overlap for these four domains between bias in randomised studies and bias in nonrandomised studies trials (although signalling questions are somewhat different in several places, see Sterne, Hernán, et al., 2016 and Higgins et al., 2019).

Randomised study outcomes are rated on a “Low/Some concerns/High” scale on each domain; whereas nonrandomised study outcomes are rated on a “Low/Moderate/Serious/Critical/No Information” scale on each domain. The level “Critical” means: the study (outcome) is too problematic in this domain to provide any useful evidence on the effects of intervention and it is excluded from the data synthesis. The same critical level of risk of bias (excluding the result from the data synthesis) is not directly present in the RoB 2 tool, according to the guidance to the tool (Higgins et al., 2019).

In the case of a RCT, where there is evidence that the randomisation has gone wrong or is no longer valid, we will assess the risk of bias of the outcome measures using ROBINS‐I instead of ROB 2. Examples of reasons for assessing RCTs using the ROBINS‐I tool may include studies showing large and systematic differences between treatment conditions while not explaining the randomisation procedure adequately suggesting that there was a problem with the randomisation process; studies with large scale differential attrition between conditions in the sample used to estimate the effects; or studies selectively reporting results for some part of the sample or for only some of the measured outcomes. In such cases, differences between the treatment and control conditions are likely systematically related to other factors than the intervention and the random assignment is, on its own, unlikely to produce unbiased estimates of the intervention effects. Therefore, as ROBINS‐I allow for an assessment of, for example, confounding, we believe it is more appropriate to assess effect sizes from studies with a compromised randomisation using ROBINS‐I than ROB 2. If so, we will report this decision as part of the risk of bias assessment of the outcome measure in question. As other effect sizes assessed with ROBINS‐I, these effect sizes may receive a “Critical” rating and thus be excluded from the data synthesis.

We will stop the assessment of a nonrandomised study outcome as soon as one domain in the ROBINS‐I is judged as “Critical”.

“Serious” risk of bias in multiple domains in the ROBINS‐I assessment tool may lead to a decision of an overall judgement of “Critical” risk of bias for that outcome and it will be excluded from the data synthesis.

##### Confounding

An important part of the risk of bias assessment of nonrandomised studies is consideration of how the studies deal with confounding factors. Systematic baseline differences between groups can compromise comparability between groups. Baseline differences can be observable (e.g., age and gender) and unobservable (to the researcher; e.g., motivation and “ability”). There is no single nonrandomised study design that always solves the selection problem. Different designs represent different approaches to dealing with selection problems under different assumptions, and consequently require different types of data. There can be particularly great variations in how different designs deal with selection on unobservables. The “adequate” method depends on the model generating participation, that is, assumptions about the nature of the process by which participants are selected into a programme.

A major difficulty in estimating causal effects of service learning on student outcomes is the potential endogeneity of service learning stemming from the decision process of introducing service learning as a pedagogical method. Not only do families choose neighbourhoods and schools, but principals and other administrators assign students to classrooms and teachers. Because these decision makers utilise information on students, teachers and schools, information that is often not available to researchers, the estimators are quite susceptible to biases from a number of sources.

As there is no universal correct way to construct counterfactuals for nonrandomised designs, we will look for evidence that identification is achieved, and that the authors of the primary studies justify their choice of method in a convincing manner by discussing the assumption(s) leading to identification (the assumption(s) that make it possible to identify the counterfactual). Preferably the authors should make an effort to justify their choice of method and convince the reader that the only difference between a treated student and a nontreated student is the treatment. The judgement is reflected in the assessment of the confounder unobservables in the list of confounders considered important at the outset (see Appendix *User guide for unobservables*).

In addition to unobservables, we have identified the following observable confounding factors to be most relevant: age and grade level, performance at baseline, gender and socioeconomic background. In each study, we will assess whether these factors have been considered, and in addition we will assess other factors likely to be a source of confounding within the individual included studies.

##### Importance of pre‐specified confounding factors

The motivation for focusing on age and grade level, performance at baseline, gender and socioeconomic background is given below.

Generally, development of cognitive functions relating to school performance and learning are age dependent, and furthermore systematic differences in performance level often refer to systematic differences in preconditions for further development and learning of both cognitive and social character (Piaget, 2002; Vygotsky, 1978).

Therefore, to be sure that an effect estimate is a result from a comparison of groups with no systematic baseline differences it is important to control for the students' grade level (or age) and their performance at baseline (e.g., reading level, math level).

With respect to gender it is well‐known that there exist gender differences in school performance (Holmlund & Sund, 2005). Girls outperform boys with respect to reading and boys outperform boys with respect to mathematics (Stoet & Geary, 2013). Although part of the literature finds that these gender differences have vanished over time (Hyde & Linn, 1988; Hyde et al., 1990), we find it important to include this potential confounder.

Students from more advantaged socioeconomic backgrounds on average begin school better prepared to learn and receive greater support from their parents during their schooling years (Ehrenberg et al., 2001). Further, Willms and Somers (2001) found that schools enroling students from higher socioeconomic backgrounds tended to have better infrastructures, more instructional materials, and better libraries. Finally, as outlined in the background section, students with socio‐economically disadvantaged backgrounds perform poorly in school tests (OECD, 2010). Therefore, the accuracy of the estimated effects of service learning will depend crucially on how well socioeconomic background is controlled for. Socioeconomic background factors are, for example, parents' educational level, family income, minority background, and so forth.

##### Effect of primary interest and important co‐interventions

We are mainly interested in the effect of starting and adhering to the intended intervention, that is, the treatment on the treated (TOT) effect. The risk of bias assessments will therefore be in relation to this specific effect. The risk of bias assessments of both randomised trials and nonrandomised studies will consider adherence and differences in additional interventions (“co‐interventions”) between intervention groups.

Important co‐interventions we will consider are interventions performed in school, during the regular school year, which are complementary to regular classes and school activities. They may be delivered individually (e.g., the Reading Apprenticeship programme or individual computer‐based training such as CogMed), in class (e.g., paired reading interventions or the Xtreme Reading programme), or in group sessions (e.g., the READ 180 programme).

##### Assessment

At least two review authors will independently assess the risk of bias for each relevant outcome from the included studies. Any disagreements will be resolved by a third reviewer with content and statistical expertise and will be reported. We will report the risk of bias assessment in risk of bias tables for each included study outcome in the completed review.

#### 3.3.5 Measures of treatment effect

##### Continuous outcomes

For continuous outcomes, effects sizes with 95% confidence intervals will be calculated, where means and standard deviations are available. If means and standard deviations are not available, we will calculate standardised mean differences (SMDs) from *F* ratios, *t* values, *χ* ^2^ values and correlation coefficients, where available, using the methods suggested by Lipsey and Wilson (2001). If not enough information is yielded, the review authors will request this information from the principal investigators. Hedges' *g* will be used for estimating SMD. Any standardised measures of student academic achievement (e.g., reading and math), are examples of relevant continuous outcomes in this review.

##### Dichotomous outcomes

For dichotomous outcomes, we will calculate odds ratios with 95% confidence intervals. Drop out and NEET status, are examples of relevant dichotomous outcomes in this review.

There are statistical approaches available to re‐express dichotomous and continuous data to be pooled together (Sánchez‐Meca et al., 2003). In order to calculate common metric odds ratios will be converted to SMD effect sizes using the Cox transformation. We will only transform dichotomous effect sizes to SMD if appropriate, for example, as may be the case with, for example, the outcomes attendance and alcohol use, that can be measured with binary and continuous data.

When effect sizes cannot be pooled, study‐level effects will be reported in as much detail as possible. Software for storing data and statistical analyses will be RevMan 5.0, Excel, R and Stata 10.0.

#### 3.3.6 Unit of analysis issues

Criteria for determination of independent findings

We will take into account the unit of analysis of the studies to determine to whether individuals were randomised in groups (i.e., cluster‐randomised trials), whether individuals may have undergone multiple interventions, whether there were multiple treatment groups and whether several studies are based on the same data source.

##### Clustered assignment of treatment

Cluster randomised trials included in this review will be checked for consistency in the unit of allocation and the unit of analysis, as statistical analysis errors can occur when they are different. When appropriate analytic methods have been used, we will meta‐analyse effect estimates and their standard errors (Higgins & Green, 2011). In cases where study investors have not applied appropriate analysis methods that control for clustering effects, we will estimate the intra‐cluster correlation (Donner et al., 2001; Hedges, 2007b) and correct standard errors.

##### Multiple interventions groups and multiple interventions per individuals

Studies with multiple intervention groups with different individuals will be included in this review, although only intervention and control groups that meet the eligibility criteria will be used in the data synthesis. To avoid problems with dependence between effect sizes we will apply robust standard errors (Hedges et al., 2010) and use the small sample adjustment to the estimator itself (Tipton, 2015). We will use the results in Tanner‐Smith and Tipton (2014) and Tipton (2015) to evaluate if there are enough studies for this method to consistently estimate the standard errors. See Section Data Synthesis below for more details about the data synthesis.

If there are not enough studies, we will use a synthetic effect size (the average) in order to avoid dependence between effect sizes. This method provides an unbiased estimate of the mean effect size parameter but overestimates the standard error. Random effects models applied when synthetic effect sizes are involved actually perform better in terms of standard errors than do fixed effects models (Hedges, 2007a). However, tests of heterogeneity when synthetic effect sizes are included are rejected less often than nominal.

If pooling is not appropriate (e.g., the multiple interventions and/or control groups include the same individuals), only one intervention group will be coded and compared to the control group to avoid overlapping samples. The choice of which estimate to include will be based on our risk of bias assessment. We will choose the estimate that we judge to have the least risk of bias (primarily, Confounding bias and in case of equal scoring the Missing outcome data domain will be used).

##### Multiple studies using the same sample of data

In some cases, several studies may have used the same sample of data or some studies may have used only a subset of a sample used in another study. We will review all such studies, but in the meta‐analysis we will only include one estimate of the effect from each sample of data. This will be done to avoid dependencies between the “observations” (i.e., the estimates of the effect) in the meta‐analysis. The choice of which estimate to include will be based on our risk of bias assessment of the studies. We will choose the estimate from the study that we judge to have the least risk of bias (primarily, Confounding bias). If two (or more) studies are judges to have the same risk of bias and one of the studies (or more) uses a subset of a sample used in another study (or studies) we will include the study using the full set of participants.

##### Multiple time points

When the results are measured at multiple time points, each outcome at each time point will be analysed in a separate meta‐analysis with other comparable studies taking measurements at a similar time point. As a general guideline, these will be grouped together as follows: (1) 0–1 year follow up, (2) 1–2 year follow up and (3) More than 2 year follow up. However, should the studies provide viable reasons for an adjusted choice of relevant and meaningful duration intervals for the analysis of outcomes, we will adjust the grouping.

#### 3.3.7 Dealing with missing data

If not enough information is yielded to calculate an effect size and standard error, the review authors will request this information from the principal investigators.

#### 3.3.8 Assessment of heterogeneity

Heterogeneity among primary outcome studies will be assessed with *χ* ^2^ (Q) test, and the *I* ^2^, and *τ* ^2^ statistics (Higgins et al., 2003). Any interpretation of the *χ* ^2^ test will be made cautiously on account of its low statistical power.

#### 3.3.9 Assessment of reporting biases

Reporting bias refers to both publication bias and selective reporting of outcome data and results. Here, we state how we will assess publication bias.

We will use funnel plots for information about possible publication bias if we find sufficient studies (Higgins & Green, 2011). However, asymmetric funnel plots are not necessarily caused by publication bias (and publication bias does not necessarily cause asymmetry in a funnel plot). If asymmetry is present, we will consider possible reasons for this.

#### 3.3.10 Data synthesis

The proposed project will follow standard procedures for conducting systematic reviews using meta‐analysis techniques.

The overall data synthesis will be conducted where effect sizes are available or can be calculated, and where studies are similar in terms of the outcome measured. Analysis of absolute effects (comparing service learning to treatment as usual) and relative effects (comparing service learning to an alternative treatment) will be conducted separately. Meta‐analysis of outcomes will be conducted on each metric (as outlined in Section 3.1.4) separately.

As different computational methods may produce effect sizes that are not comparable, we will be transparent about all methods used in the primary studies (research design and statistical analysis strategies) and use caution when synthesising effect sizes. Special caution will be taken concerning studies using regression discontinuity designs (RDD) to estimate the treatment effect. In sharp RDDs, a threshold of a (nonmanipulable) forcing/running variable determines which students receive a treatment and which do not, that is, the design is similar to a RCT in the sense that the random sequence determining treatment assignment can be seen as a running variable (Lee & Lemieux, 2010). In contrast, in “fuzzy” RDDs, being on one side of a threshold only makes it more likely that a student end up in the treatment or control group, and the threshold is used as an instrument to estimate local average treatment effects (LATE) (Angrist & Pischke, 2009; Imbens & Lemieux, 2008). That is, fuzzy RDD is a form of IV analysis, which we will exclude due to the comparability issues mentioned earlier. Sharp RDDs will be included, but, as the effects may be estimated on a very “local” sample close to a threshold, may be subject to a separate analysis depending on the comparability to samples from other studies. We will in any case check the sensitivity of our results to the inclusion of RDD studies. In addition, we will discuss the limitation in generalisation of results obtained from these types of studies.

When the effect sizes used in the data synthesis are odds ratios, they will be log transformed before being analysed. The reason is that ratio summary statistics all have the common feature that the lowest value that they can take is 0, that the value 1 corresponds with no intervention effect, and the highest value that an odds ratio can ever take is infinity. This number scale is not symmetric. The log transformation makes the scale symmetric: the log of 0 is minus infinity, the log of 1 is zero, and the log of infinity is infinity.

Studies that have been coded with a critical risk of bias will not be included in the data synthesis.

As the intervention deal with diverse populations of participants (from different countries, facing different curriculums, etc.), and we therefore expect heterogeneity among primary study outcomes, all analyses of the overall effect will be inverse variance weighted using random effects statistical models that incorporate both the sampling variance and between study variance components into the study level weights. Random effects weighted mean effect sizes will be calculated using 95% confidence intervals and we will provide a graphical display (forest plot) of effect sizes. Graphical displays for meta‐analysis performed on ratio scales sometimes use a log scale, as the confidence intervals then appear symmetric. This is however not the case for the software Revman 5 which we plan to use in this review.^1^ The graphical displays using odds ratios and the mean effect size will be reported as a odds ratio.

For subsequent analyses of moderator variables that may contribute to systematic variations, we will use the mixed‐effects regression model. This model is appropriate if a predictor explaining some between‐studies variation is available but there is a need to account for the remaining uncertainty (Hedges & Pigott, 2004; Konstantopoulos, 2006).

We expect that several studies have used the same sample of data. We will review all such studies, but in the meta‐analysis we will only include one estimate of the effect from each sample of data. This will be done to avoid dependencies between the “observations” (i.e., the estimates of the effect) in the meta‐analysis. The choice of which estimate to include will be based on our quality assessment of the studies. We will choose the estimate from the study that we judge to have the least risk of bias, with particular attention paid to Confounding bias.

We anticipate that several studies provide results separated by, for example, age and/or gender. We will include results for all age and gender groups. To take into account the dependence between such multiple effect sizes from the same study, we will apply robust variance estimation (RVE) approach (Hedges et al., 2010). An important feature of this analysis is that the results are valid regardless of the weights used. For efficiency purposes, we will calculate the weights using a method proposed by Hedges et al. (2010). This method assumes a simple random‐effects model in which study average effect sizes vary across studies (*τ* ^2^) and the effect sizes within each study are equicorrelated (*ρ*). The method is approximately efficient, since it uses approximate inverse‐variance weights: they are approximate given that *ρ* is, in fact, unknown and the correlation structure may be more complex. We will calculate weights using estimates of *τ* ^2^, setting *ρ* = 0.80 and conduct sensitivity tests using a variety of *ρ* values; to asses if the general results and estimates of the heterogeneity is robust to the choice of *ρ*. We will use the small sample adjustment to the residuals used in RVE as proposed by Bell and McCaffrey (2002) and extended by McCaffrey et al. (2001) and by Tipton (2015). We will use the Satterthwaite degrees of freedom (Satterthwaite, 1946) for tests as proposed by Bell and McCaffrey (2002) and extended by Tipton (2015). We will use the guidelines provided in Tanner‐Smith and Tipton (2014) to evaluate if there are enough studies for this method to consistently estimate the standard errors.

If there is not a sufficient number of studies to use RVE we will conduct a data synthesis where we use a synthetic effect size (the average) in order to avoid dependence between effect sizes.

#### 3.3.11 Subgroup analysis and investigation of heterogeneity

We will investigate the following factors with the aim of explaining potential observed heterogeneity: study‐level summaries of participant characteristics (e.g., studies considering a specific gender, age or socioeconomic level or studies where separate effects for girls/boys, primary school/secondary school or low/high socioeconomic status are available) and quality of the service learning programme according to the standards as outlined in section *The intervention*. We expect that there will be limited information in many studies preventing us from measuring all eight standards. We anticipate we will be able to focus on five of them. These five relate to (a) linking programmes to academic and programme curriculum or objectives, (b) incorporating youth voice, (c) involving community partners, (d) providing opportunities for reflection and (e) duration and intensity. In the Appendix (*Data extraction* section) it is outlined how we will code these five standards.

If the number of included studies is sufficient and given there is variation in the covariates, we will perform moderator analyses (multiple meta‐regression using the mixed model) to explore how observed variables are related to heterogeneity.

If there are a sufficient number of studies, we will apply the RVE approach and use approximately inverse variance weights calculated using a method proposed by Hedges et al. (2010). This technique calculates standard errors using an empirical estimate of the variance: it does not require any assumptions regarding the distribution of the effect size estimates. The assumptions that are required to meet the regularity conditions are minimal and generally met in practice. This more robust technique is beneficial because it takes into account the possible correlation between effect sizes separated by the covariates within the same study and allows all of the effect size estimates to be included in meta‐regression. We will calculate weights using estimates of *τ* ^2^, setting *ρ* = 0.80 and conduct sensitivity tests using a variety of *ρ* values; to asses if the general results is robust to the choice of *ρ*. We will use the small sample adjustment to the residuals used in RVE and the Satterthwaite degrees of freedom (Satterthwaite, 1946) for tests (Tipton, 2015). The results in Tipton (2015) suggests that the degrees of freedom depend on not only the number of studies but also on the type of covariates included in the meta‐regression. The degrees of freedom can be small, even when the number of studies is large if a covariate is highly unbalanced or a covariate with very high leverage is included. The degrees of freedom will vary from coefficient to coefficient. The corrections to the degrees of freedom enable us to assess when the RVE method performs well. As suggested by Tanner‐Smith and Tipton (2014) and Tipton (2015) if the degrees of freedom are smaller than four, the RVE results should not be trusted.

We will report 95% confidence intervals for regression parameters. We will estimate the correlations between the covariates and consider the possibility of confounding. Conclusions from meta‐regression analysis will be cautiously drawn and will not solely be based on significance tests. The magnitude of the coefficients and width of the confidence intervals will be taken into account as well. Otherwise, single factor subgroup analysis will be performed. The assessment of any difference between subgroups will be based on 95% confidence intervals. Interpretation of relationships will be cautious, as they are based on subdivision of studies and indirect comparisons.

In general, the strength of inference regarding differences in treatment effects among subgroups is controversial. However, making inferences about different effect sizes among subgroups on the basis of between‐study differences entails a higher risk compared to inferences made on the basis of within study differences; see Oxman et al. (1992). We will therefore use within study differences where possible.

We will also consider the degree of consistence of differences, as making inferences about different effect sizes among subgroups entails a higher risk when the difference is not consistent within the studies (see Oxman et al., 1992).

#### 3.3.12 Sensitivity analysis

Sensitivity analysis will be carried out by restricting the meta‐analysis to a subset of all studies included in the original meta‐analysis and will be used to evaluate whether the pooled effect sizes are robust across components of risk of bias. We will consider sensitivity analysis for each domain of the risk of bias checklists and restrict the analysis to studies with a low risk of bias.Sensitivity analyses with regard to research design and statistical analysis strategies in the primary studies will be an important element of the analysis to ensure that different methods produce consistent results.

## TREATMENT OF QUALITATIVE RESEARCH

We do not plan to include qualitative research.

## CONTRIBUTIONS OF AUTHORS

Trine Filges is an experienced systematic reviewer and methodologist, having completed a number of systematic reviews in social welfare topic areas as well as in the field of education. She has published fifteen Campbell Systematic reviews, is currently the lead reviewer on two Campbell Systematic Reviews, further involved as a reviewer in six Campbell Systematic Reviews and an evidence gap map and has published systematic and meta‐analytic reviews in high‐impact journals. Trines fields of expertise are systematic review methods and statistical analysis; and she will contribute to the quantitative data extraction, methodological quality appraisal and meta‐analysis.

Nina Thorup Dalgaard is a psychologist, Ph.D. Nina has previously worked as an educational psychologist within a primary school setting and thus has knowledge about theories of learning and didactic and about the socioemotional and cognitive development of children within an educational setting.

Jens Dietrichson is an educationalist, experienced systematic reviewer and methodologist, having completed a number of systematic reviews in field of education and has published systematic and meta‐analytic reviews in high‐impact journals. He is currently the lead reviewer on one Campbell Systematic Review and is knowledgeable regarding all major facets of meta‐analytic methods and their application. Jens's fields of expertise are systematic review methods and statistical analysis; and he will contribute to the quantitative data extraction, methodological quality appraisal and meta‐analysis.

Bjørn C. A. Viinholt has 3 years of experience in developing and writing systematic reviews. As a part of undertaking systematic reviews, Bjørn has experience in developing systematic search strategies and processes of reference management. Bjørn will contribute with assisting and development of the systematic search strategy, reference management and grey literature searches for this review—as well as assisting with aspects relating to systematic literature searches in Campbell review methodology.

## SOURCES OF SUPPORT

### Internal sources

- VIVE Campbell, Denmark

### External sources

- No sources of support provided

## Footnotes

1 If we apply robust variance estimation, the analysis will be conducted in STATA or R as robust variance estimation is not implemented in Revman 5.

## First and second level screening

First level screening is on the basis of titles and abstracts. Second level is on the basis of full text

Reference id. No.:

Reviewers initials:

Source:

Year of publication:

Country/countries of origin:

Author(s):

The study will be excluded if one or more of the answers to Questions 1–3 are “No” If the answers to Questions 1–3 are “Yes” or “Uncertain,” then the full text of the study will be retrieved for second level eligibility. All unanswered questions need to be posed again on the basis of the full text. If not enough information is available, or if the study is unclear, the author of the study will be contacted if possible.

**Screening questions:**

1. Does the study focus on service learning?
  Yes—include
  No—if no then stop here and exclude
  Uncertain—include
  Question 1 guidance:
  The intervention in this review is service learning. Studies considering extra‐curriculum activities or stand‐alone volunteer or community activity will not be eligible.
2. Are the participants children in grades kindergarten to 12 (or the equivalent in European countries) in general education?
  Yes—include
  No—if no then stop here and exclude
  Uncertain—include
  Question 2 guidance:
  Regular private, public or boarding schools are eligible. We exclude children in home‐school. In some countries, kindergarten can however refer to preschool programmes outside of primary school and include ages down to 2 years. Service learning targeting such populations will be excluded; that is, kindergarten must be considered a part of primary school for a study to be included.
3. Is the report/article a quantitative evaluation study with a comparison condition?

Yes—include

No—if no then stop here and exclude

Uncertain—include

Question 3 guidance:

We are only interested in primary quantitative studies with a comparison group, where the authors have analysed the data. We are not interested in theoretical papers on the topic or surveys/reviews of studies of the topic. (This question may be difficult to answer on the base of titles and abstracts alone).

## Data extraction

**Table:**

**Names of author(s)**
**Title**
**Language**
**Journal**
**Year**
**Country**
**Type of school**—**private, boarding, public**
**Participant characteristic (age, grade level, gender, socioeconomic status, ethnicity)**
**Programme feature** *Linking to curriculum (answer yes or no)*, a yes requires that the study as a minimum have reported, having clear goals for the programme that align with the curriculum, and containing corresponding activities to match those goals.
**Programme feature** *Youth voice*, code yes when it is reported that students were involved in the
planning, implementation, or evaluation process of the program
**Programme feature** *Community involvement*, code yes if it is reported that the community has some part in the programme besides providing a place for students to serve
**Programme feature** *Reflection*, code yes if some type of reflection activity (e.g., using journals, having discussions in class or in small groups, writing essays about the service experience, presenting to the class what was learned, or reflecting individually with the teacher or site supervisor are reported
**Programme feature** *Duration* (number of weeks, one semester, one school year)
**Programme feature** *Intensity* (number of community service hours per week/month)
**Type of data used in study (administrative, questionnaire, other (specify))**
**Level of aggregation (individual or class)**
**Time period covered by analysis (divide into intervention and follow up)**
**Sample size (divide into treated/comparison)**

## User guide for unobservables

Systematic baseline differences between groups can compromise comparability between groups. Baseline differences can be observable (e.g., age and gender) and unobservable (to the researcher; e.g., motivation and “ability”). There is no single nonrandomised study design that always solves the selection problem. Different designs solve the selection problem under different assumptions and require different types of data. Especially how different designs deal with selection on unobservables varies. The “right” method depends on the model generating participation, that is, assumptions about the nature of the process by which participants are selected into a programme.

As there is no universal correct way to construct counterfactuals we will assess the extent to which the identifying assumptions (the assumption that makes it possible to identify the counterfactual) are explained and discussed (preferably the authors should make an effort to justify their choice of method). We will look for evidence that authors using, for example (this is NOT an exhaustive list):

**Natural experiments:**

Discuss whether they face a truly random allocation of participants and that there is no change of behaviour in anticipation of, for example, policy rules.

**Matching (including propensity scores):**

Explain and discuss the assumption that there is no selection on unobservables, only selection on observables.

**(Multivariate, multiple) regression:**

Explain and discuss the assumption that there is no selection on unobservables, only selection on observables. Further discuss the extent to which they compare comparable people.

**Regression discontinuity (RD):**

Explain and discuss the assumption that there is a (strict!) RD treatment rule. It must not be changeable by the agent in an effort to obtain or avoid treatment. Continuity in the expected impact at the discontinuity is required.

**Difference‐in‐difference (treatment‐control‐before‐after):**

Explain and discuss the assumption that the trends in treatment and control groups would have been parallel, had the treatment not occurred.

## Justification of exclusion of studies using an instrumental variable (IV) approach

Studies using instrument variables (IV) for causal inference in nonrandomised studies will not be included as the interpretation of IV estimates is challenging. IV only provides an estimate for a specific group namely, people whose behaviour change due to changes in the particular instrument used. It is not informative about effects on never‐takers and always‐takers because the instrument does not affect their treatment status. The estimated effect is thus applicable only to the subpopulation whose treatment status is affected by the instrument. As a consequence, the effects differ for different IVs and care has to be taken as to whether they provide useful information. The effect is interesting when the instrument it is based on is interesting in the sense that it corresponds to a policy instrument of interest. Further, if those that are affected by the instrument are not affected in the same way the IV estimate is an average of the impacts of changing treatment status in both directions, and cannot be interpreted as a treatment effect. To turn the IV estimate into a LATE requires a monotonicity assumption. The movements induced by the instrument go in one direction only, from no treatment to treatment. The IV estimate, interpreted as a LATE, is only applicable to the complier population, those that are affected by the instrument in the “right way”. It is not possible to characterise the complier population as an observation's subpopulation cannot be determined and defiers do not exist by assumption.

In the binary‐treatment–binary‐instrument context, the IV estimate can, given monotonicity, be interpreted as a LATE; that is, the average treatment effect for the subpopulation of compliers. If treatment or instruments are not binary, interpretation becomes more complicated. In the binary‐treatment–multivalued‐instrument (ordered to take values from 0 to *J*) context, the IV estimate, given monotonicity, is a weighted average of pairwise LATE parameters (comparing subgroup *j* with subgroup *j* − 1). The IV estimate can thus be interpreted as the weighted average of average treatment effects in each of the *J* subgroups of compliers. In the multivalued‐treatment (ordered to take values from 0 to *T*)—multivalued‐instrument (ordered to take values from 0 to *J*) context, the IV estimate for *each pair of instrument values*, given monotonicity, is a weighted average of the effects from going from *t* − 1 to *t* for persons induced by the change in the value of the instrument to move from any level below *t* to the level *t* or any level above. Persons can be counted multiple times in forming the weights.
